# Dual species dynamic transcripts reveal the interaction mechanisms between *Chrysanthemum morifolium* and *Alternaria alternata*

**DOI:** 10.1186/s12864-021-07709-9

**Published:** 2021-07-09

**Authors:** Lina Liu, Fadi Chen, Sumei Chen, Weimin Fang, Ye Liu, Zhiyong Guan

**Affiliations:** State Key Laboratory of Crop Genetics and Germplasm Enhancement, Key Laboratory of Landscaping, Ministry of Agriculture and Rural Affairs, Key Laboratory of Biology of Ornamental Plants in East China, College of Horticulture, National Forestry and Grassland Administration, Nanjing Agricultural University, 210095 Nanjing, China

**Keywords:** dual RNA-seq, *Chrysanthemum morifolium*, *Alternaria alternata*, plant-pathogen interaction, WGCNA

## Abstract

**Background:**

Chrysanthemum (*Chrysanthemum morifolium*) black spot disease caused by *Alternaria alternata* is one of the plant’s most destructive diseases. Dual RNA-seq was performed to simultaneously assess their transcriptomes to analyze the potential interaction mechanism between the two species, i.e., host and pathogen.

**Results:**

*C. morifolium* and *A. alternata* were subjected to dual RNA-seq at 1, 12, and 24 h after inoculation, and differential expression genes (DEGs) in both species were identified. This analysis confirmed 153,532 DEGs in chrysanthemum and 14,932 DEGs in *A. alternata*, which were involved in plant-fungal interactions and phytohormone signaling. Fungal DEGs such as toxin synthesis related enzyme and cell wall degrading enzyme genes played important roles during chrysanthemum infection. Moreover, a series of key genes highly correlated with the early, middle, or late infection stage were identified, together with the regulatory network of key genes annotated in the Plant Resistance Genes database (PRGdb) or Pathogen-Host Interactions database (PHI-base). Highly correlated genes were identified at the late infection stage, expanding our understanding of the interplay between *C. morifolium* and *A. alternata*. Additionally, six DEGs each from chrysanthemum and *A. alternata* were selected for quantitative real-time PCR (qRT-PCR) assays to validate the RNA-seq output.

**Conclusions:**

Collectively, data obtained in this study enriches the resources available for research into the interactions that exist between chrysanthemum and *A. alternata*, thereby providing a theoretical basis for the development of new chrysanthemum cultivars with resistance to pathogen.

**Supplementary Information:**

The online version contains supplementary material available at 10.1186/s12864-021-07709-9.

## Background

Chrysanthemum, one of the most commercially important ornamental crops worldwide, is widely used as cut flowers, potted plants, and in landscaping. It carries a long history of cultivation, high ornamental, edible, and medicinal value [1]. Chrysanthemum is susceptible to pathogen invasion during cultivation, especially when grown on a large scale. Alternaria leaf spot is a major disease of chrysanthemum that readily occurs at high temperatures and during continuous rainy seasons. Following a symptomless early infection stage, small round black spots form at the *A. alternata* invasion site, which eventually expand into round, round-like, or irregular spots covered with a dark mildew layer [2]. Currently, the main method of *A. alternata* control in chrysanthemum is via fungicide application. However, a prolonged use of these chemicals can result in pathogen resistance and environmental pollution. Thus, a better understanding of the defense mechanisms employed by chrysanthemum in response to *A. alternata* will help design new and safer control strategies, as well as develop resistant cultivars. By performing dual RNA-seq analysis on chrysanthemum and *A. alternata* simultaneously, we can understand changes in transcriptional expression related to chrysanthemum defense against *A. alternata*. Furthermore, we can determine which *A. alternata* genes interact with chrysanthemum and analyze the molecular response of *A. alternata*-infected plants.

In response to external biotic stress, plants induce a range of immune responses, including production of physical barriers (e.g., keratin, wax, lignin, and special stomatal structures) [3], chemical barriers (e.g., secondary metabolites with antibacterial properties) [4], and molecular responses (e.g., hypersensitive response, production of reactive oxygen species, and expression of pathogen-related genes) [5]. High-throughput sequencing technology, especially RNA-seq, tracks more precise molecular changes in plants under biotic and abiotic stress. This method has been widely applied in research on plant-pathogen interactions in agricultural crops, including in apple (*Malus* × *domestica*) [6], citrus [7], gape (*Vitis vinifera*) [8], pear (*Pyrus pyrifolia*) [9], soybean [10], and tomato (*Solanum lycopersicum*) [11]. Interactions between hosts and *A. alternata* were also investigated by RNA sequencing [2, 9, 12]. In a recent study, a model was conducted to elucidate the response of chrysanthemum leaves to *A. alternata* infection at different stages[2], which laid a foundation for the further research on the interaction between chrysanthemum and *A. alternata*. These previous investigations showed a complex interaction between host and *A. alternata*. Above all, ethylene (ET) signal transduction pathway, calcium signal transduction pathway, and plant-pathogen interaction pathway all were involved in the response to *A. alternata* infection in *C. morifolium*. Based on the above studies, we speculate that there is a complex interaction between chrysanthemum and pathogens. However, most studies above were limited to a unilateral transcription analysis of *C. morifolium* under pathogenic stress, but the mutual attack and counterattack response between chrysanthemum and pathogen are poorly understood. More recently, dual RNA-seq has become a powerful tool for comprehensively understanding host-pathogen interactions in vivo [13], that can simultaneously capture pathogen-specific transcripts during the infection process, provide a more complete view of interactions [14], reveal biosynthetic and metabolic pathways of crosstalk among participants, and specifically determine the dynamic expression profile of genes associated with host-pathogen interactions [15]. To date, the mutual in vivo attack and counterattack response between chrysanthemum and *A. alternata* are poorly understood. The present study aimed to investigate *C. morifolium* infected with *A. alternata* using dual RNA-seq analysis.

RNA-seq libraries were constructed and identified DEGs were further analyzed. The expression of fungal genes were also investigated at three infection stages, in an attempt to discover genes that could potentially threaten the cultivation of chrysanthemum. Besides, qRT-PCR assays were carried out to verify the reliability of the dual RNA-seq data by gene primers listed in Table S1. Through the study, we hoped to gain insights into the interaction between *C. morifolium* and *A. alternata*, and to investigate the potential pathogenesis of *A. alternata*, as well as the defense mechanism of *C. morifolium*, which would benefit in inhibiting fungal pathogenicity or breed resistant chrysanthemum cultivars.

## Results

### Statistical analysis of RNA-seq results

**Fig. 1:**
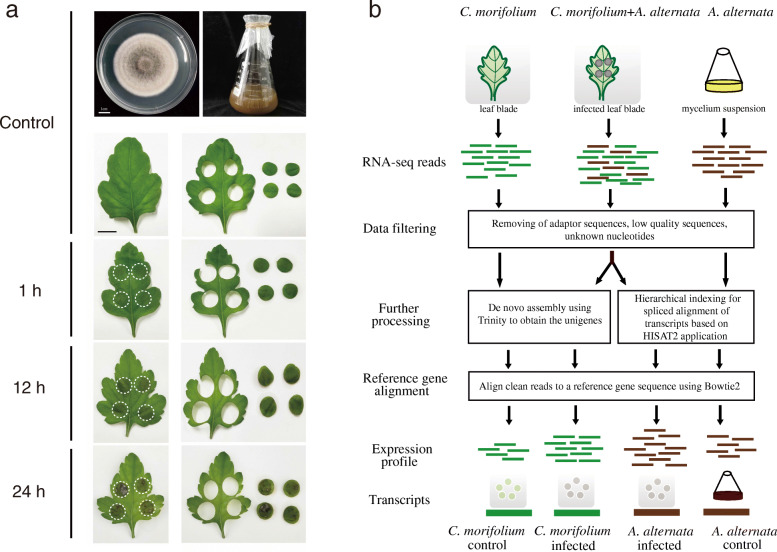
Inoculating, sampling, and dual RNA-seq analysis process. **a** Morphology of *A. alternata*, healthy chrysanthemum leaves, and *A. alternata*-inoculated chrysanthemum leaves. Image showing leaf spots on the upper side at 1, 12, and 24 HPI. Scale bar = 1 cm; **b** Flow chart representing dual RNA-seq analysis of mixed transcriptome obtained from chrysanthemum leaves infected with *A. alternata*

*A. alternata* morphology, symptom changes in inoculated chrysanthemum leaves, and the dual RNA-seq analysis process are shown in Fig. 1. Three samples sets, each with three biological replicates, were subjected to dual RNA-seq at each time point, and 27 cDNA libraries were generated: CK1h_1, CK1h_2, CK1h_3, CK12h_1, CK12h_2, CK12h_3, CK24h_1, CK24h_2, CK24h_3, Aa1h_1, Aa1h_2, Aa1h_3, Aa12h_1, Aa12h_2, Aa12h_3, Aa24h_1, Aa24h_2, Aa24h_3, In1h_1, In1h_2, In1h_3, In12h_1, In12h_2, In12h_3, In24h_1, In24h_2, In24h_3 (CK: control treatment, only chrysanthemum leaves; In: inoculation treatment, contain chrysanthemum leaves and *A. alternata* mycelium; Aa: only *A. alternata* mycelium). Table S2 lists the summary statistics of original reads and filtered clean reads obtained from three replicates at each time point, for mapping to the reference genome of *A. alternata*. The average clean reads of the inoculated and control samples generated on average 108.93 Mb and 108.64 Mb clean reads, respectively, with a read ratio ≥ 92.54 %. Moreover, at each time point, inoculated and control samples contained an average of 10.89 Gb and 10.86 Gb of clean bases, respectively. Table S3 shows the summary statistics of original reads and filtered clean reads of three replicates at each time point, for chrysanthemum. Inoculated and control samples of chrysanthemum generated on average 41.20 Mb and 108.76 Mb clean reads, respectively, all with 100 % read ratio. Furthermore, at each time point, inoculated and control samples contained average 4.12 Gb clean bases and 10.88 Gb clean bases, respectively.

The clustered quality indicators of chrysanthemum are shown in Table S4. Infected and control chrysanthemum samples contained an average of 35,843 and 54,560 unigenes, respectively. The total length of chrysanthemum library transcripts was ≥ 14,571,366, the average length of the library was ≥ 642, and N50, N70, and N90 ≥ 865, 556, 296, respectively. The GC ratio was ≥ 40.45 %. Comparison of all unigenes to the seven major functional databases for annotation, generated the following numerical data: 89,889 (NR: 72.62 %), 55,679 (NT: 44.98 %), 61,156 (SwissProt: 49.41 %), 64,694 (KOG: 52.26 %), 64,705 (KEGG: 52.27 %), 68,727 (GO: 55.52 %), and 60,671 (Pfam: 49.01 %) (Table S5). The average total mapping percentage of *A. alternata* at each time point was higher than 56.9 %, and the control group was higher than 86.53 % (Table S6). The reads that mapped to *A. alternata* were also mapped to the chrysanthemum, and the total mapping rate is lower than 0.20 % (Table S7). Due to the low mapping rate to chrysanthemum, the reads mapped to *A. alternata* were considered unique to *A. alternata*.

### Identification of DEGs

**Fig. 2:**
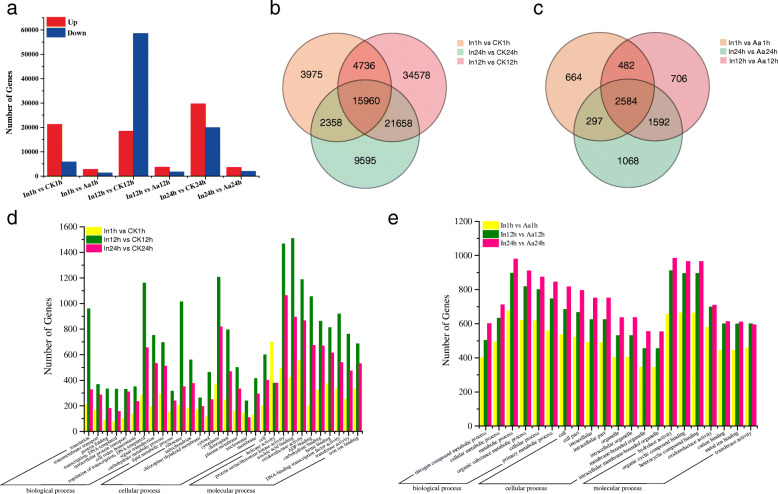
DEGs comparisons between sample types. **a** Number of DEGs compared between two sample types (i.e., In1h vs. CK1h, In1h vs. Aa1h, In12h vs. CK12h, In12h vs. Aa12h, In24h vs. CK24h, and In24h vs. Aa24h). DEGs are shown in red (up-regulated) and blue (down-regulated); **b** Venn diagram representation of unique and shared DEGs between the ‘In’ and ‘CK’ sample series, at the tested time points; **c** Venn diagram representation of unique and shared DEGs between the ‘In’ and ‘Aa’ sample series, at the tested time points; **d** GO functional enrichment analysis of chrysanthemum DEGs; **e** GO functional enrichment analysis of *A. alternata* DEGs

Comparison of gene expression between the ‘In’ and ‘CK’ sample series detected 27,029 DEGs (21,216 up-regulated and 5,813 down-regulated) for In1h vs. CK1h, 76,932 DEGs (18,446 up-regulated and 58,486 down-regulated) for In12h vs. CK12h, and 49,571 DEGs (29,642 up-regulated and 19,929 down-regulated) for In24h vs. CK24h (Fig. 2a). Illustration of these results as a Venn diagram clearly showed that both unique and shared DEGs were identified between, and among times points (Fig. 2c). For example, 18,318, 20,696, and 37,618 shared DEGs were detected in the 1 HPI (hours post inoculation) vs. 24 HPI, 1 HPI vs. 12 HPI, and 12 HPI vs. 24 HPI comparisons, respectively, while 15,960 DEGs were found in the 1 HPI vs. 12 HPI vs. 24 HPI comparison (Fig. 2b). These results suggested that, as pathogen infection progressed, an increasing number of genes became involved in defense responses.

The degree of GO term enrichment was similar for the three inoculation time points, and DEGs were divided into 54 functional categories according to biological processes (25), cellular components (16), and molecular functions (13). The most significantly enriched biological processes were “regulation of transcription, DNA-templated”, “carbohydrate metabolic process”, and “translation”; the most significantly enriched cellular components were “cytoplasm”, “ribosome”, and “chloroplast”, while “protein serine/threonine kinase activity”, “nucleic acid binding”, and “oxidoreductase activity” occupied the important positions in molecular functions (Fig. 2d). Moreover, a total of 30 KEGG pathways were significantly enriched at 1, 12, and 24 HPI, each with a varying number of DEGs (Table S8). Maps with the highest DEG representation were those for ‘plant-pathogen interactions’ (ko 04626), followed by those for ‘plant hormone signal transduction’ (ko 04075), ‘MAPK signaling pathway-plant’ (ko 04016), ‘carbon metabolism’ (ko 01200) ‘protein processing in endoplasmic reticulum’ (ko 04141), and ‘biosynthesis of amino acids’ (ko01230). The above results indicated that chrysanthemum infected with *A. alternata* involved a series of defense strategies interacting with multiple pathways to jointly regulate and respond to pathogenic stress. These strategies dominated at different infection stages.

Gene expression comparison between the ‘In’ and ‘Aa’ sample series found 4,027 DEGs (2,729 up-regulated and 1,298 down-regulated) for In1h vs. Aa1h, 5364 DEGs (3697 up-regulated and 1667 down-regulated) for In12h vs. Aa12h, and 5,541 DEGs (3572 up-regulated and 1969 down-regulated) for In24h vs. Aa24h (Fig. 2a). Moreover, analysis of Venn diagram showed 2,881, 3,066, 4,176, and 2,584 shared DEGs in the 1 HPI vs. 24 HPI, 1 HPI vs. 12 HPI, 12 HPI vs. 24 HPI, and 1 HPI vs. 12 HPI vs. 24 HPI comparisons (Fig. 2c).

The degree of GO term enrichment was similar among the three stages of *A. alternata* infection, and DEGs were divided into 38 functional categories, according to biological processes (16), cellular components (12), and molecular functions (10). “Metabolic process”, “organic substance metabolic process”, and “cellular process” were the most significantly enriched biological processes; the most significantly enriched cellular components were detected in “cell”, “cell part”, and “intracellular”; while “hydrolase activity”, “organic cyclic compound binding”, and “heterocyclic compound binding” occupied the important positions in molecular functions (Fig. 2e). Moreover, a total of 20 KEGG pathways were significantly enriched at the three stages, but with varying numbers of DEGs (Table S9). Maps with the highest DEGs representation were for ‘biosynthesis of antibiotics’ (ko 01130), followed by ‘MAPK signaling pathway-yeast’ (ko 04011), ‘amino sugar and nucleotide sugar metabolism’ (ko 00520), and ‘glycine, serine and threonine metabolism’ (ko 00260). The above results indicated that *A. alternata* induced a variety of metabolic activities during chrysanthemum infection, which generated energy and toxic metabolites to attack host cells. These metabolic processes played a key role in the interaction between chrysanthemum and *A. alternata*.

### DEGs involved in phytohormone signaling

**Fig. 3:**
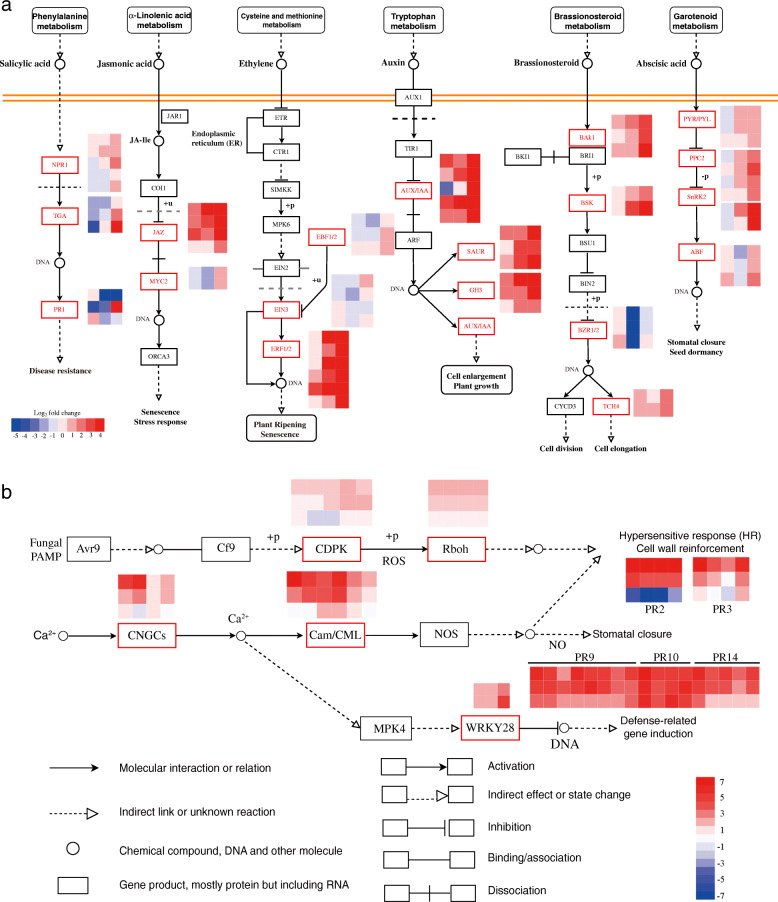
DEGs involved in phytohormone signaling transduction and plant-fungal interaction pathways. **a** DEGs involved in phytohormone signaling transduction pathways. From left to right: salicylic acid (SA), jasmonic acid (JA), ethylene (ET), auxin (AUX), brassinosteroid (BR), and abscisic acid (ABA). Each vertical column represents 1, 12, and 24 HPI from left to right, and each horizontal row represents a DEG; **b** DEGs involved in plant-fungal interactions. Each vertical column represents a DEG and each horizontal row represents 1, 12, and 24 HPI from bottom to top. Expression values are presented as log_2_ fold-change value (red represents up-regulation; blue represents down-regulation)

Phytohormones, such as salicylic acid (SA), ET, jasmonic acid (JA), brassionosteroid (BR), auxin (AUX), and abscisic acid (ABA), are widely involved, and play critical regulatory roles in plant-pathogen interactions [16]. The related DEGs of several hormone signaling pathways in infected chrysanthemum leaves were analyzed. Several DEGs involved in SA biosynthesis and signaling were differentially expressed, e.g., three DEGs of *NPR1* homologues and *TGA* homologues were down-regulated at 1 HPI but significantly up-regulated at 24 HPI; two DEGs homologous to *PR1* were up-regulated at 1 HPI, and one of them was up-regulated at 24 HPI. All DEGs homologous to *JAZ* were up-regulated during the whole process, and those *MYC2* homologous were significantly up-regulated at 24 HPI. Several genes known to be ET-responsive were up-regulated, including *EBF1/2* homologues at 24 HPI, *EIN3* homologues at 1 HPI and 24 HPI, and *ERF1/2* homologues, which exhibited change by a higher multiple. Most DEGs in AUX signaling, such as *AUX*/*IAA*, *SAUR* and auxin-responsive *GH3* homologues also showed notably up-regulated expression. Previous studies also shown that BR comprises a unique class of growth-promoting steroid hormones, known to be key regulators of plant immunity [17]. DEGs encoding BR signaling cascades included *BAK1*, *BSK*, *TCH4*, and *BZR1*/*2*. Except for DEGs homologous to *BZR1*/*2*, that responded to *A. alternata* at 1 HPI, but were down-regulated by a high multiple at 12 HPI; the remaining DEGs belonging to the BR signaling cascades, expression level gradually increased at three infection stages. Finally, DEGs involved in ABA signaling pathway, such as *PYR*/*PYL*, *PP2C*, and *SnRK2* homologues, were all up-regulated at 24 HPI; *ABF* homologue was up-regulated at 1 HPI and 24 HPI, but down-regulated at 12 HPI, like *BZR1*/*2* homologue. The schematic diagram of the relevant hormone pathways is shown in Fig. 3a.

### DEGs involved in plant-fungal interaction

During biotic stress, chrysanthemum DEGs encoding CDPK (calcium-dependent protein kinase) and Rbohs (respiratory explosive oxidase homologs) were significantly up-regulated, which were involved in hypersensitive reaction (HR) and cell wall reinforcement. DEGs encoding Pathogenesis-related (PR) proteins, such as chitinase (*PR3*), were generally up-regulated, while the DEGs encoding β-1,3-glucanase (*PR2*) were specially down-regulated at 1 HPI, but significantly up-regulated at 12 HPI, with a high multiple (> 6) notably induced at 24 HPI. In addition, most DEGs encoding potential cyclic nucleotide gated channels (CNGCs) were up-regulated at 24 HPI, and those encoding CaM/CMLs showed similar trends. Furthermore, several downstream defense-related PR genes, such as *PR9* (peroxidase), *PR10* (ribonuclease), and *PR14* (lipid-transfer protein), were induced and significantly up-regulated at three time points (Fig. 3b).

### DEGs related to virulence in ***A. alternata***

**Fig. 4:**
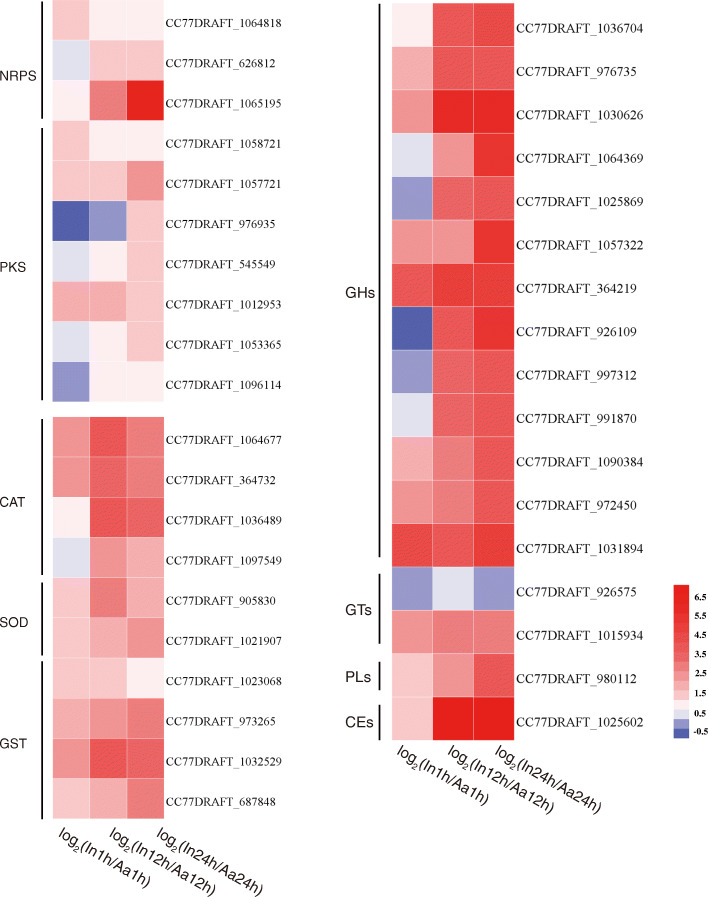
DEGs related to *A. alternata* virulence

*Alternaria* spp. produce a variety of secondary metabolites during the pathogenic process, and more than 70 compounds with significant toxicity had been isolated [18], with important roles in fungal virulence. Most of these toxins are versatile compounds of polyketides and non-ribosomal peptides, which are usually generated by *NRPS* and *PKS*, respectively [19]. We identified three *NRPS* and seven *PKS* homologous genes in *A. alternata*, all showing up-regulated expression at three inoculation stages. *NRPS* homologue (*CC77DRAFT_1065195*) presented a significantly higher multiple (> 6) at 24 HPI (Fig. 4). A previous study had shown that *pksJ* and *pksH* were correlated with the production of alternariol and alternariol-9-methyl ether [20]. We also identified the *pksJ* homolog (*CC77DRAFT_1058721*) and *pksH homolog* (*CC77DRAFT_976935*), both were up-regulated during *A. alternata* infection (Fig. 4). Besides, *A. alternata* also expressed some genes involved in detoxification and stress tolerance. Among them, DEGs encoding catalase peroxidase (CAT), superoxide dismutase (SOD), and glutathione S-transferase (GST) were all significantly up-regulated, which are essential for pathogens to respond to host defenses (Fig. 4).

Furthermore, fungal cell wall degrading enzymes (CAZymes) can promote degradation of the plant cell wall, penetration into the host tissue, and adhesion layer formation [21]. CAZymes consist of four functional classes: glycoside hydrolases (GHs), glycosyl transferases (GTs), polysaccharide lyases (PLs), and carbohydrate esterases (CEs), classified according to their catalytic modules or functional domains [21]. Expression levels were also investigated during the interaction between *A. alternata* and chrysanthemum. There were thirteen DEGs of GHs, with most of them significantly up-regulated at the three time points; two DEGs of GTs, one up-regulated and the other down-regulated. Only one DEG of PLs was found, and its expression showed an obviously upward trend. Like PLs, only one CE displayed higher expression (Fig. 4).

### Weighted gene co-expression network analysis (WGCNA)

**Fig. 5:**
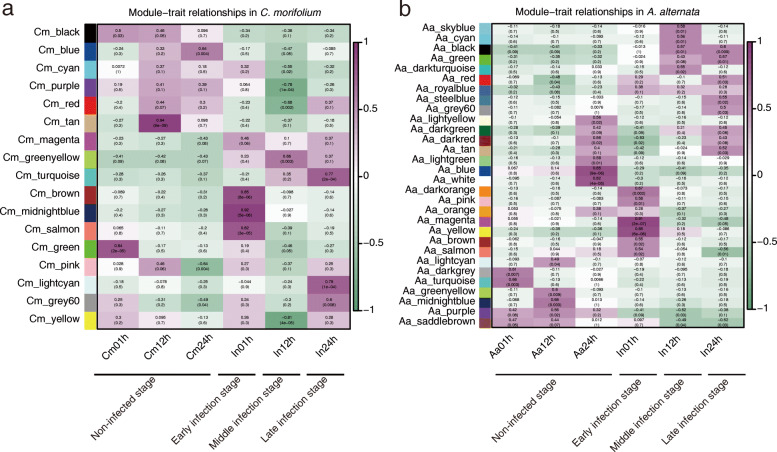
WGCNA results revealed modules highly correlated with phenotype traits in *C. morifolium* (**a**) and *A. alternata* (**b**)

Weighted gene co-expression network analysis (WGCNA) was carried out to identify genes related to phenotypes and investigated the co-expression networks to elucidate the interaction network between *C. morifolium* and *A. alternata*. Ultimately, 17 and 29 gene co-expression modules were discovered in *C. morifolium* and *A. alternata*, respectively, shown in Fig. 5a and b.

Genes from the ‘Cm_brown’, ‘Cm_midnightblue’, ‘Cm_salmon’, ‘Cm_greenyellow’, ‘Cm_lightcyan’, ‘Aa_magenta’, ‘Aa_yellow’, ‘Aa_brown’, ‘Aa_skyblue’ and ‘Aa_black’ modules were highly correlated with the traits observed at three infection stages (Fig. 5a, b). KEGG annotation analyses were performed to further explore what pathways the genes from the modules above were involved in. Plant cell walls can act as a natural physical barrier against pathogens [21]. The cuticle is the first layer of the cell wall that prevents pathogens from invading the cells [22], and usually consists of a horny and a waxy protective film. Its biosynthesis involves several genes, including wax-ester synthase/diacylglycerol O-acyltransferase (WSD) [23], and fatty acid omega-hydroxy dehydrogenase (HTH) [24]. In the ‘Cm_turquoise’ module, *WSD* and *HTH* homologues in *C. morifolium* were significantly up-regulated, and several of these gene homologues (e.g., *Unigene36512_All*, *Unigene36513_All*, and *CL1059.Contig1_All*) displayed an expression fold-change > 10 (Figure S1). The above analysis showed that the cuticle played a positive role in the chrysanthemum defense against *A. alternata*. As reported, pectin lyase, pectate lyase, and xylanase can break down the pectin and xylans present in the plant cell wall. In the ‘Aa_black’ and ‘Aa_green’ modules, lyase homologues (e.g., *CC77DRAFT_1043109*, *CC77DRAFT_1048882*, and *CC77DRAFT_167134*) exhibited a high expression level in *A. alternata* during infection (Figure S1).

### Highly correlated modules and key genes identification

**Fig. 6:**
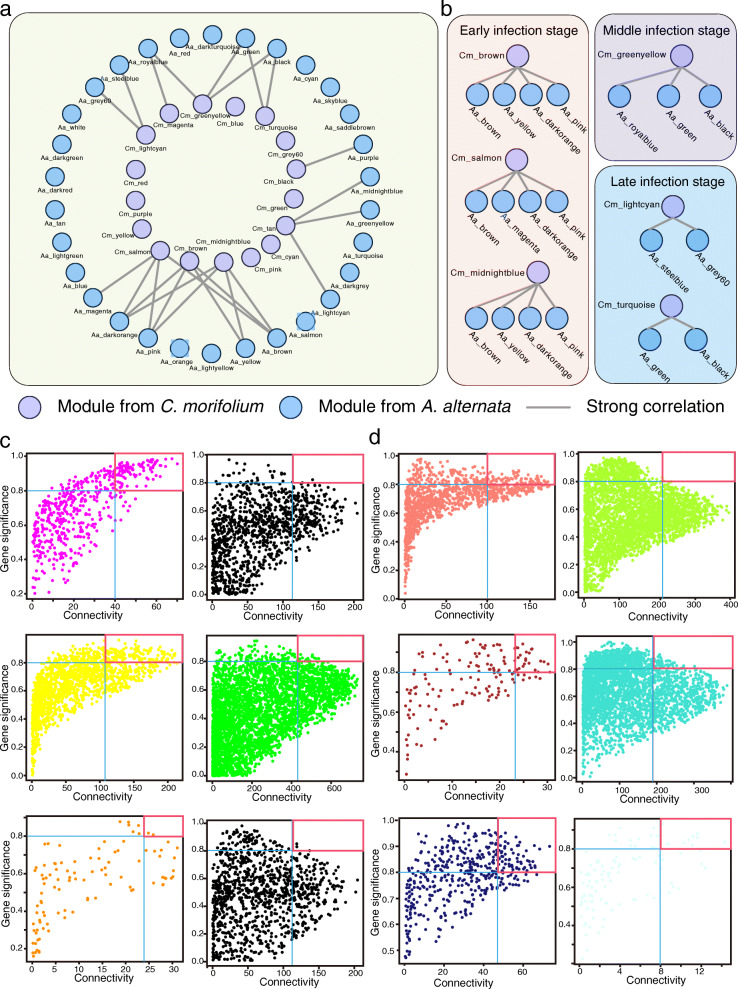
Correlation analysis between modules from *C. morifolium* and *A. alternata. ***a** Network of *C. morifolium* and *A. alternata* modules. Highly correlated modules (*r* ≥ 0.8 and *p*-value < 0.05) are linked by a line; **b** Network of *C. morifolium* and *A. alternata* modules at different stages of infection; **c**, **d** Key gene identification in the highly correlated modules of *C. morifolium* (**c**) and *A. alternata* (**d**)

The relationship between module and trait allowed us to evaluate the correlation coefficient between modules from *C. morifolium* and *A. alternata*. A network of *C. morifolium* and *A. alternata* modules were shown in Fig. 6a, and highly correlated modules (r ≥ 0.8 and p-value < 0.05) were linked by a line (Fig. 6a). In the early infection stage, three *C. morifolium* gene modules (‘Cm_brown’, ‘Cm_midnightblue’, and ‘Cm_salmon’) and five *A. alternata* modules (‘Aa_magenta’, ‘Aa_yellow’, ‘Aa_brown’, ‘Aa_darkorange’, and ‘Aa_pink’) were highly correlated; in the middle infection stage, one *C. morifolium* gene module (‘Cm_greenyellow’) and three *A. alternata* modules (‘Aa_royalblue’, ‘Aa_black’, and ‘Aa_green’) were highly correlated; in the late infection stage, two *C. morifolium* gene modules (‘Cm_turquoise’ and ‘Cm_lightcyan’) and four *A. alternata* modules (‘Aa_steelblue’, ‘Aa_grey60’, ‘Aa_black’, and ‘Aa_green’) were highly correlated (Fig. 6b). Gene significance (value ≥ 0.8) and connectivity (top 20 %) were used together to identify key genes in each of the modules above (Fig. 6c, d).

**Fig. 7:**
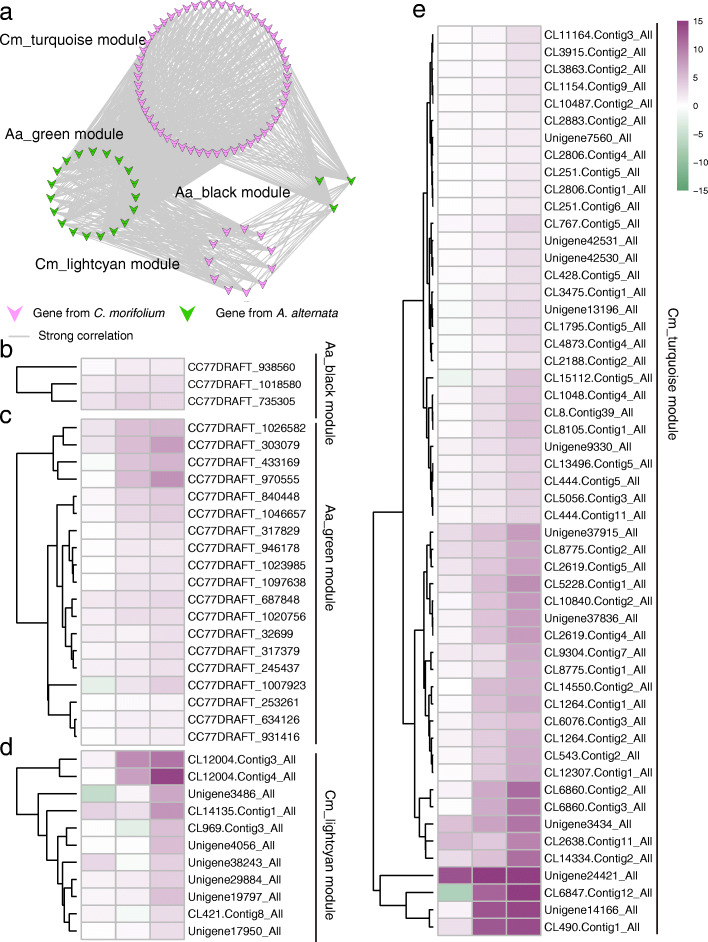
Gene interplay between *C. morifolium* and *A. alternata* at the late infection stage. **a** Network genes from *C. morifolium* and *A. alternata* in the late infection stage. Highly correlated genes (*r* ≥ 0.8 and *p*-value < 0.05) are linked by a line; **b-e.** Heatmap of highly correlated genes from *C. morifolium* and *A. alternata* at the late infection stage

Based on gene transcription level, we performed correlation coefficient analyses between genes from highly correlated modules in *C. morifolium* and *A. alternata* to identify the interplay genes. From modules that highly correlated with the late infection stage, several genes were identified and a network of highly correlated genes (r ≥ 0.8 and p-value < 0.05) were linked by a line, as shown in Fig. 7a. Most of these genes were up-regulated with the spread of *A. alternata* (Fig. 7b, c, d, e).

### Regulatory network of key genes annotated in PPI and PRG databases

**Fig. 8:**
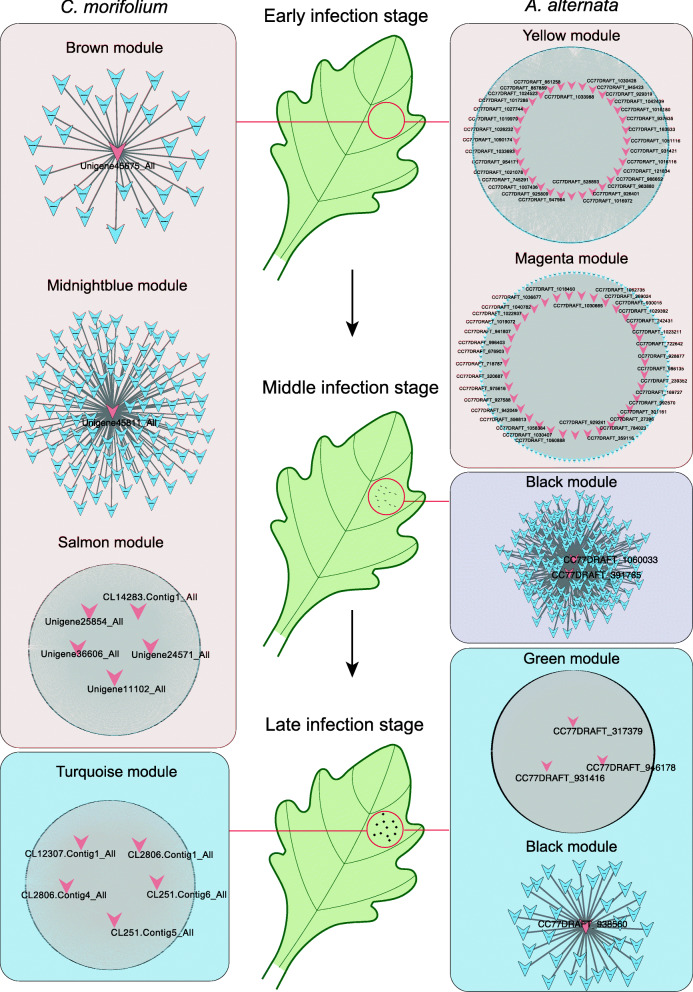
Co-expression networks of key genes from *C. morifolium* and *A. alternata* at the three stages of infection

PRGdb (http://prgdb.org) is a bioinformatics platform for plant resistance gene analysis [25]. PHI-base (www.phi-base.org) contains molecular and biological information on genes which had been proven to affect the outcome of host-pathogen interactions [26]. Key genes from the highly correlated modules were examined for further analyses. Seventy-five key genes from *A. alternata* were annotated by PHI-base and twelve key genes were annotated by PRGdb. The regulatory network of these eighty-seven key genes was shown in Fig. 8. Notably, two disease resistance genes, RGA1-like homologs (*CL2806.Contig1_All* and *CL2806.Contig4_All*), were identified in the late infection stage. Two transcription factors (*CL14283.Contig1_All* and *Unigene25854_All*) were also identified, and may play important roles in response to *A. alternata* infection in *C. morifolium* (Fig. 8). Additionally, *ACL2* homolog (*CC77DRAFT_784023*), *ACL1* homolog (*CC77DRAFT_986135*), and *BUF1* homolog (*CC77DRAFT_528893*) were identified, which were predicted to influence the virulence of *A. alternata* (Fig. 8).

### Validation of RNA-seq data by qRT-PCR

**Fig. 9:**
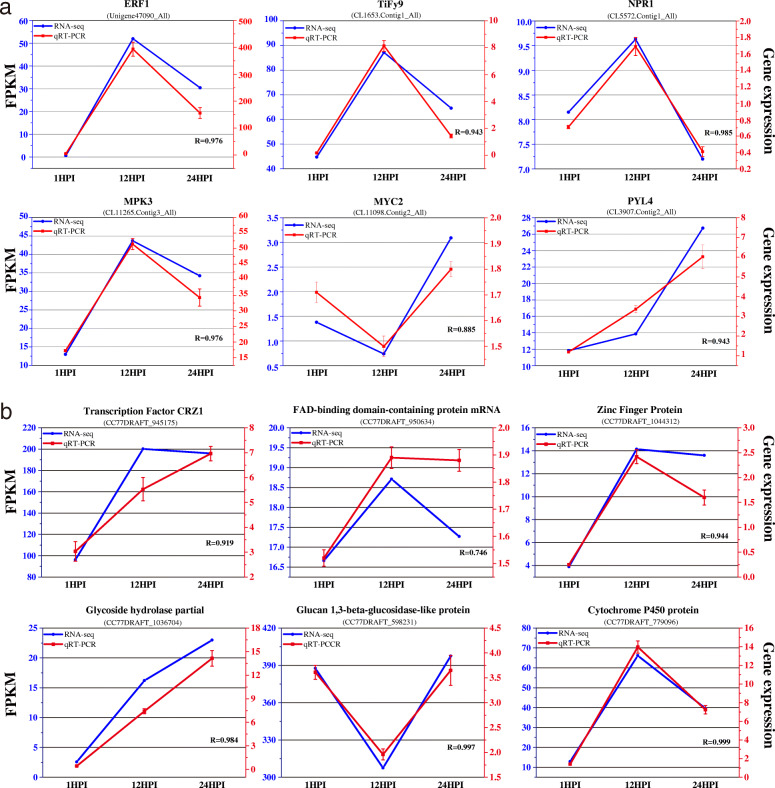
The relative expression level change of DEGs from *C. morifolium* (**a**) and *A. alternata* (**b**) by RNA-seq and qRT-PCR. Left vertical axis coordinate represents FPKM from RNA-seq (blue); right vertical axis coordinate represents relative gene expression level from qRT-PCR (red). R-values are the relative coefficients between qRT-PCR and RNA-seq

To confirm the reliability of the generated dual RNA-seq data, the expression of 12 DEGs were analyzed using qRT-PCR assays, of which six were derived from chrysanthemum, (*CL11098.Contig2_All*, *CL1653.Contig1_All*, *CL5572.Contig1_All*, *Unigene47090_All*, *CL3907.Contig2_All* and *CL11265.Contig3_All*; Fig. 9a), and six were from *A. alternata* (*CC77DRAFT_945175*, *CC77DRAFT_1044312*, *CC77DRAFT_1036704*, *CC77DRAFT_598231*, *CC77DRAFT_779096* and *CC77DRAFT_950634*; Fig. 9b). The qRT-PCR results and RNA-seq data showed similar up-regulation or down-regulation expression patterns. The correlation coefficients between qRT-PCR and RNA-seq of the 12 DEGs were all ≥ 0.85. Minor discrepancies regarding the expression levels might suggest a difference in sensitivity between the two methods. These results highlighted the reliability of the RNA-seq data.

## Discussion

Dual RNA-seq of chrysanthemum leaves infected with *A. alternata* was performed to detect the occurrence of any dynamic changes in the plant tissue, which would provide a broader understanding of the mechanism of host-pathogen interaction between the two species. This study compared the gene expression of *A. alternata*, chrysanthemum leaves, and chrysanthemum leaves infected with *A. alternata* at three infection stages, i.e., the early (no lesion formation), middle (lesion formation), and late (lesion expansion) infection stage. A total of 153,532 and 14,932 DEGs were identified in chrysanthemum and *A. alternata*, respectively. The analysis of these DEGs focused on induced pathways in chrysanthemum or *A. alternata* during infection.

Many DEGs of chrysanthemum were enriched in the “Plant-pathogen interaction” pathway. DEGs encoding CDPK and Rbohs were also identified, that were accompanied by ROS accumulation during infection, resulting in HR and cell wall enhancement. Several enzyme systems had been reported to characterize oxidative bursts of HR. For instance, ascorbic acid (ASC) can act in coordination with glutathione (GSH) and other important enzymatic antioxidants in the AsA-GSH cycle to provide an appropriate redox environment required to regulate various defense pathways, such as the expression of defense genes through activation of the *NPR1* regulatory transcription factor, strengthening of cell walls, and modulation of defense-hormonal signal networks [27]. Significant up-regulation of DEGs encoding ASC and GSH were also detected in the generated data (Figure S2), suggesting that the ASC and GSH systems may be induced as part of a transduction pathway that triggered defense responses and sequential cell death. Calmodulin plays a significant role in sensing and transducing changes in cellular Ca^2+^ concentration in response to several biotic and abiotic stresses [28]. During the interaction between *C. morifolium* and *A. alternata*, a series of defensive signals were also activated, including DEGs encoding CaM/CMLs, which were significantly up-regulated. In addition, the chitinase can hydrolyze the chitin component of the pathogen cell wall, and release elicitors for defense responses [29]. Activities of the two chitinases in infected chrysanthemum leaves were significantly higher than control leaves, highlighting their importance in defending against *A. alternata* in *C. morifolium*.

Plant hormones play important roles in regulating developmental processes and signaling networks involved in the plant’s response to a wide range of biotic and abiotic stresses [30]. ET signaling components, such as *EIN2*, *EIN3*, *EBF1/2*, *ERF1/2*, are involved in the regulation of cell death and defense responses [31]. JA signaling is systemically activated in response to various biotic and abiotic stresses, increasing the resistance of host plants to some pathogens [32]. SA also plays an important role in resistance and defense induction in response to pathogen attacks [33]. In this study, more than twenty DEGs involved in ET, JA, and SA metabolism were significantly up-regulated at 24 HPI. Their interplay induced defense responses to *A. alternata* infection. In the present study, several DEGs associated with ABA and BR signaling were up-regulated in the chrysanthemum response to *A. alternata* infection, suggesting that BR and ABA could be participants in this regulatory response. The participation and characteristics of DEGs in complex phytohormone signaling pathways indicate that these signals were not only simple linear and isolated cascades, but also cooperated with one another in response to *A. alternata* infection.

Most importantly, chrysanthemum developed a series of immunity responses when inoculated with *A. alternata*, during which time the pathogen secreted effectors to suppress the host plant’s immunity response. Several *A. alternata* genes, beneficial to the pathogen’s infection and colonization were also significantly induced during infecting chrysanthemum leaves. The tangerine pathotype of *A. alternata* produces host-selective ACT-toxin, the biosynthesis of which is essentially encoded by a polyketide synthase gene that is also required for pathogenicity of this fungus [34]. *NRPS* and cytochrome P450 protein *TES1* are required for tentoxin biosynthesis in *A. alternata* strain ZJ33 [35], while the *PKS* gene *ACRTS2* is responsible for host-selective ACR-Toxin biosynthesis in the rough lemon pathotype of *A. alternata* [36]. In the present study, DEGs corresponding to *NRPS* and *PKS* homologs were also identified, confirming the importance of this toxin synthesis during *A. alternata* invasion into chrysanthemum. Several studies had also demonstrated that effector proteins can affect plant immune mechanisms by regulating plant gene transcription [37], affecting the secretion of and degrading plant immune-related proteins [38–41], affecting the connection of host cell walls and cell membranes [42, 43], and regulating plant hormone synthesis and related signaling pathways [44–46]. Extracellular degrading enzymes produced by plant pathogenic fungi are important types of fungal effectors [47]. Our research revealed that a series of degrading enzyme gene homologs were up-regulated, which may be investigated in the future to elucidate the pathogenic mechanism of *A. alternata*.

To further determine the interaction mechanism between *C. morifolium* and *A. alternata* during the different (early, middle, and late) infection stage, WGCNA and correlation coefficient analysis were carried out. A series of highly correlated modules between *C. morifolium* and *A. alternata* were identified. PRGdb is a bioinformatics platform for plant resistance gene analysis [25], and PHI-base contains molecular and biological information on genes that have been proven to affect the outcome of pathogen-host interactions [26]. The regulatory network of key genes annotated in PRGdb or PHI-base at three infection stages were visualized using the Cytoscape software. For example, the regulatory networks of two disease resistance genes, *RGA1*-like homologs, were identified at the late infection stage. The *ACL1*, *ACL2*, and *BUF1* homologs were also identified, which were predicted to influence the virulence of *A. alternata*. Transcription factors are important players in the response to pathogen invasion [48, 49]. The regulatory network of two transcription factors (*CL14283.Contig1_All* and *Unigene25854_All*) were also identified, which may play important roles in response to *A. alternata* infection in *C. morifolium*. Moreover, using the correlation coefficient between key genes of *C. morifolium* and *A. alternata*, highly correlated genes were identified, reinforcing our understanding of the interplay between the two species.

Currently, the interaction mechanism between chrysanthemum and *A. alternata* is not fully understood, and the function of effector proteins from *A. alternata* are unknown too. The discovery of *A. alternata* toxin synthesis genes and candidate effectors will not only improve our understanding of *A. alternata* pathogenesis, but also perhaps more significantly, provide valuable resources for subsequent investigations into plant-pathogen interactions. The present study has designed a powerful methodology for mixed transcriptome analysis of host plant and pathogen, which has established a foundation for comprehensive research on the pathogenesis of chrysanthemum black spot disease.

## Conclusions

In the study, *A. alternata*, chrysanthemum leaves, and chrysanthemum leaves infected with *A. alternata* at three infection stages, i.e., the early (no lesion formation), middle (lesion formation), and late (lesion expansion) infection stages were sampled for dual RNA-seq. A total of 153,532 and 14,932 DEGs were identified in chrysanthemum and *A. alternata*, respectively. Chrysanthemum employed multiple pathways to jointly regulate and respond to pathogenic stress. *A. alternata* induced a variety of metabolic activities during infection, that generated energy and toxic metabolites to attack host cells. The discovery of *A. alternata* toxin synthesis genes and candidate effectors will not only improve our understanding of *A. alternata* pathogenesis, but also perhaps more significantly, provide valuable resources for subsequent investigations into plant-pathogen interactions. Meanwhile, WGCNA and correlation coefficient analysis were carried out to identify the regulatory network of key genes from highly correlated modules at the three infection stages. Coefficient analyses showed that several genes were highly correlated between *C. morifolium* and *A. alternata* at the late infection stage, which provide a broader understanding of the interaction mechanisms between two species. This work gains insights into the interaction between *C. morifolium* and *A. alternata* and elucidate the potential pathogenesis of *A. alternata*, as well as the defense mechanism of *C. morifolium*, which would benefit in inhibiting fungal pathogenicity or breed resistant chrysanthemum cultivars.

## Methods

### Plant materials and ***A. alternata*** culture

Chrysanthemum cultivar ‘Dayangju’ was obtained from the Chrysanthemum Germplasm Resource Preserving Centre of Nanjing Agricultural University, China. Rooting seedlings of approximately similar growth were transplanted into a mixed matrix of 3:1 vermiculite and perlite without add fertilizer. Growth was under 16 h photoperiod, day and night temperatures set to 25 ℃ and 22 ℃ respectively, and relative humidity maintained at 68–75 % [1, 2]. The test strain *A. alternata* was isolated and identified from typical diseased leaves of ‘Fubaiju’, a cultivar found in the chrysanthemum tea producing area of Futianhe Town, Macheng City, Hubei Province, China in 2017. And *A. alternata* was stored in 15 % v/v glycerol, and held in a freezer at − 80 °C. The test strain was transferred to plates containing PDA (Potato Dextrose Agar) solid medium on a sterile bench, and cultured at 25 ℃ before inoculation assays.

### ***A. alternata*** inoculation and sampling

Inoculation assays were performed as previously described [2]. The strain *A. alternata* was cultured in 200 mL of PDW (Potato Dextrose Water) liquid medium on a 200 r/min shaker for 24 h. Then, 1 mL homogenous mycelium suspension was collected (the amount of mycelium contained in each milliliter of suspension was constant), used a fine-bristle brush to pick out the mycelium to inoculate it on four positions on the leaves (up, down, left, and right on leaves). Each plant was inoculated with two leaves, and each leaf was inoculated with four inoculation sites as shown in Fig. 1a. And every inoculation site was round, about 1 cm in diameter. The above procedure can ensure that each site was inoculated with a quantitative amount of mycelium. The treatment and control groups were cultured in an incubator maintained at 28 ℃ and 90 % humidity in the dark. Once the inoculated leaves reached 1, 12, and 24 HPI, representing the three infection stages, the groups were sampled simultaneously. Leaf blocks in each sample were taken from different leaves. Specifically, at each inoculation time point, in the inoculated group, a total of 48 small leaf blocks were collected from 6 plants, and 10 small leaf blocks were randomly selected from them constituted 1 biological replicate. A total of 3 times selections constituted 3 biological replicates. The control group (without fungus) were similarly sampled in corresponding areas of chrysanthemum leaves. During chrysanthemum sampling at the three time points, *A. alternata* mycelium was simultaneously sampled on a clean bench. Samples of inoculated and control chrysanthemum leaves, as well as *A. alternata* mycelium were all collected in three replicates, frozen in liquid nitrogen and stored at − 80 °C for dual RNA-seq.

### RNA extraction, library construction, and sequencing

Total RNA was isolated from each sample using the RNA-iso Plus reagent (TaKaRa Bio, Tokyo, Japan) following the manufacturer’s protocol. To assess the integrity, the concentration was tested using a Nano Drop spectrophotometer (Thermo Fisher Scientific, Waltham, MA, USA), and the quality was tested using the Agilent 2100 Bio analyzer (Agilent Technologies, Santa Clara, CA, USA) to include RIN value, 28 S/18S ratio, and fragment length distribution. mRNA was enriched using magnetic beads with Oligo (dT); the RNA was fragmented, and reverse-transcribed to double-stranded cDNA using N6 random primers. The synthesized cDNA was subjected to end-repair followed by 3′ adenylation, and adaptors were ligated to the ends of these 3′ adenylated cDNA fragments. The ligation products were purified, and PCR amplification was performed to enrich the purified cDNA template, using PCR primers. Lastly, the amplicons were denatured by heat, and single-stranded DNA was cyclized using splint oligos and DNA ligase. The generated libraries were then used for sequencing on the BGISEQ-500 platform, and the products labelled as ‘raw reads’ [50]. Twenty-seven sets of original readings were obtained, corresponding to control chrysanthemum leaf samples (CK1h, CK12h, CK24h; hereafter named the ‘CK’ sample series), *C. morifolium* leaves infected with *A. alternata* (In1h, In12h, In24h; hereafter named the ‘In’ sample series), *A. alternata* (Aa1h, Aa12h, Aa24h; hereafter named the ‘Aa’ sample series), and with three replicates for per sample.

### Raw reads mapping and functional annotation

After sequencing, the raw data of all the samples were filtered to remove low quality reads, including adaptor sequences, low quality sequences, and unknown nucleotides, and obtain clean reads. After filtering, clean reads were compared to *A. alternata* genome. After removing the data that is determined to be the *A. alternata* genome, the remaining data were regarded as the clean data of chrysanthemum. Read ratio is the percentage of clean reads of the total reads used for transcriptome analysis of every species. As the reference genome of chrysanthemum was unpublished, the remaining data defaulted to the chrysanthemum part data were used for the de novo assembly to get the chrysanthemum reference sequence. Firstly, all generated raw sequencing reads were filtered using the SOAPnuke software, to remove low quality reads, including adaptor sequences, low quality sequences (where the percentage of low-quality bases with a value ≤ 10 was more than 20 % in one read), and unknown nucleotides (where unknown bases were more than 5 %), and obtain clean reads. Secondly, clean reads with overlap joints were combined to form longer fragments, i.e., contigs. Finally, clean reads were assembled using Trinity (v2.0.6), and transcripts were clustered using TGICL, to remove redundancy, and obtain unigenes for functional annotation. In the case of multiple samples, TGICL was used again to perform clustering on each sample’s unigenes to remove redundancy and obtain the final unigenes for subsequent analysis [51]. Clean reads were aligned to a reference gene sequence using Bowtie2, and the expression level of the unigene was calculated via the FPKM (fragments per kilobase of transcript per million fragments mapped) method [52]. DEGs were defined according to a threshold of Q-values ≤ 0.001 [53] and an absolute log_2_ ratio value ≥ 1, among the three biological replicates. Sequences were compared with the NR (http://ncbi.nlm.nih.gov/blast/db), NT (http://ncbi.nlm.nih.gov/blast/db), Swiss-Prot (www.uniprot.org), Pfam (http://pfam.xfam.org), KEGG (http://www.genome.jp/kegg), KOG (https://www.ncbi.nlm.nih.gov/COG/), and GO (http://geneontology.org) databases, in order to identify and annotate the generated DEGs [54, 55]. GO categories were assigned to all genes via a BLASTX hit using the Blast2GO software. KEGG was used to map sequences to pathways, and the KOBAS [56] software was used to test the statistical enrichment of DEGs identified in the KEGG pathways. Functions with a Q-value ≤ 0.05 were generally considered to be significantly enriched. Transcription factor prediction was determined by using getorf (http://emboss.sourceforge.net/apps/cvs/emboss/apps/getorf.html) to find each DEG’s ORF, which was then aligned to TF domains (from PlntfDB) using hmmsearch (http://hmmer.org) [57]. As the *A. alternata* genome was published, clean reads (obtained as described above) were aligned to reference genome sequences (https://www.ncbi.nlm.nih.gov/genome/11201?genome_assembly_id=275364) by hierarchical indexing for spliced alignment of transcripts in the HISAT (Hierarchical Indexing for Spliced Alignment of Transcripts) application [58]. The reads mapped to *A. alternata* were also mapped to chrysanthemum by HISAT software [58] to measure whether the reads above were unique to *A. alternata*. *A. alternata* DEGs were identified using a method like that described for chrysanthemum. The DIAMOND software (https://github.com/bbuchfink/diamond) was used to annotate the DEG comparison to the PHI-base, and annotation results were further screened based on conditions where query coverage ≥ 50 % and identity ≥ 40 %, in order to find potentially pathogenic genes in *A. alternata*. At the same time, GO classification and KEGG pathway enrichment were also performed [59].

### Weighted gene co-expression network analysis

Weighted gene co-expression network analysis (WGCNA) was performed to identify key genes using the WGCNA R package [60]. The adjacency matrix was built based on normalized FPKM values, following which modules containing transcripts with similar expression patterns were created, and key genes for these modules were calculated. Gene significance (value > = 0.8) and connectivity (top 20 %) were used to identify hub genes. Co-expression networks were visualized using Cytoscape software [61]. Highly correlated modules and genes were calculated by correlation coefficient, and defined according to a threshold of r ≥ 0.8 and a p-value < 0.05.

### qRT-PCR validation and analysis

RNA-seq results were validated by selecting 12 DEGs to examine the consistency of their expression profiles. Total RNA (1 mg) was reverse transcribed using the Prime Script™ RT Master Mix (Perfect Real Time) (Takara) following the manufacturer’s instructions. Gene-specific primers for qRT-PCR analysis were designed using the Primer 5.0 software. The chrysanthemum *CmEF1α* gene was used as a reference, and gene primers were listed in Table S1. Three biological replicates were performed per sample, and qRT-PCR was performed as previously described by Li et al. [1]. The relative expression level of each sample was calculated using the 2^−∆∆CT^ method [62].

## Supplementary Information

**Additional file 1:** **Figure S1 **Heatmap of genes involved in cell wall reinforcement and disassembly. Expression values are presented as log_2_ fold-change value (red represents up-regulation; blue represents down-regulation).

**Additional file 2:** **Figure S2 **Heatmap of genes involved in ascorbic acid and glutathione synthesis. Expression values are presented as log_2_ fold-change value (red represents up-regulation; blue represents down-regulation).

**Additional file 3:** **Table S1 **Primer sequences used in qRT-PCR for the validation of dual RNA-seq data.

**Additional file 4:** **Table S2 **Summary statistics of raw reads and clean reads used for mapping to reference genome of *A. alternata*.

**Additional file 5:** **Table S3 **Summary statistics of chrysanthemum clean reads.

**Additional file 6:** **Table S4 **Summary of the assembly results of chrysanthemum.

**Additional file 7:** **Table S5** Unigene annotation overview of chrysanthemum.

**Additional file 8:** **Table S6** Summary of clean read mapping to *A. alternata* genomic database.

**Additional file 9:** **Table S7** Mapping rate of clean read mapped to *A. alternata* to chrysanthemum.

**Additional file 10:Table S8** Results of KEGG pathway enrichment analysis of chrysanthemum.

**Additional file 11:** **Table S9** Results of KEGG pathway enrichment analysis of *A. alternata*.

## Data Availability

The sequencing datasets generated during the current study are available in the NCBI Sequence Read Archive repository under accession number PRJNA725278. *A. alternata* genome used in the study is from the website https://www.ncbi.nlm.nih.gov/genome/11201?genome_assembly_id=275364.

## Abbreviations

DEGs: Differential expression genes
CK: Control treatment, only chrysanthemum leaves
In: Inoculation treatment, contain chrysanthemum leaves and *A. alternata* mycelium
Aa: Only *A. alternata* mycelium
SA: Salicylic acid
ET: Ethylene
JA: Jasmonic acid
BR: Brassionosteroid
AUX: Auxin
ABA: Abscisic acid
CDPK: Calcium-dependent protein kinase
Rbohs: Respiratory explosive oxidase homologs
HR: Hypersensitive reaction
PR: Pathogenesis-related
CNGCs: Cyclic nucleotide gated channels
CAZymes: Cell wall degrading enzymes
GHs: Glycoside hydrolases
GTs: Glycosyl transferases
PLs: Polysaccharide lyases
CEs: Carbohydrate esterases
WGCNA: Weighted gene co-expression network analysis
WSD: Wax-ester synthase/diacylglycerol O-acyltransferase
HTH: Fatty acid omega-hydroxy dehydrogenase
PRGdb: The Plant Resistance Genes database
PHI: Base:the Pathogen-Host Interactions database
ASC: Ascorbic acid
GSH: Glutathione
PDA: Potato Dextrose Agar
PDW: Potato Dextrose Water
HPI: Hours post inoculation
FPKM: Fragments per kilobase of transcript per million fragments mapped
HISAT: Hierarchical Indexing for Spliced Alignment of Transcripts
qRT-PCR: Quantitative real-time PCR
CAT: Catalase peroxidase
SOD: Superoxide dismutase
GST: Glutathione S-transferase
