# Supplementary material for: Dual species dynamic transcripts reveal the interaction mechanisms between Chrysanthemum morifolium and Alternaria alternata
Source: BMC Genomics. 2021 Jul 9;22:523. doi: 10.1186/s12864-021-07709-9 (PMC8268330; doi:10.1186/s12864-021-07709-9)
Supplement: Supplementary file 1 — Additional file 1: Figure S1 Heatmap of genes involved in cell wall reinforcement and disassembly. Expression values are presented as log2 fold-change value (red represents up-regulation; blue represents down-regulation). [file 12864_2021_7709_MOESM1_ESM.pdf]

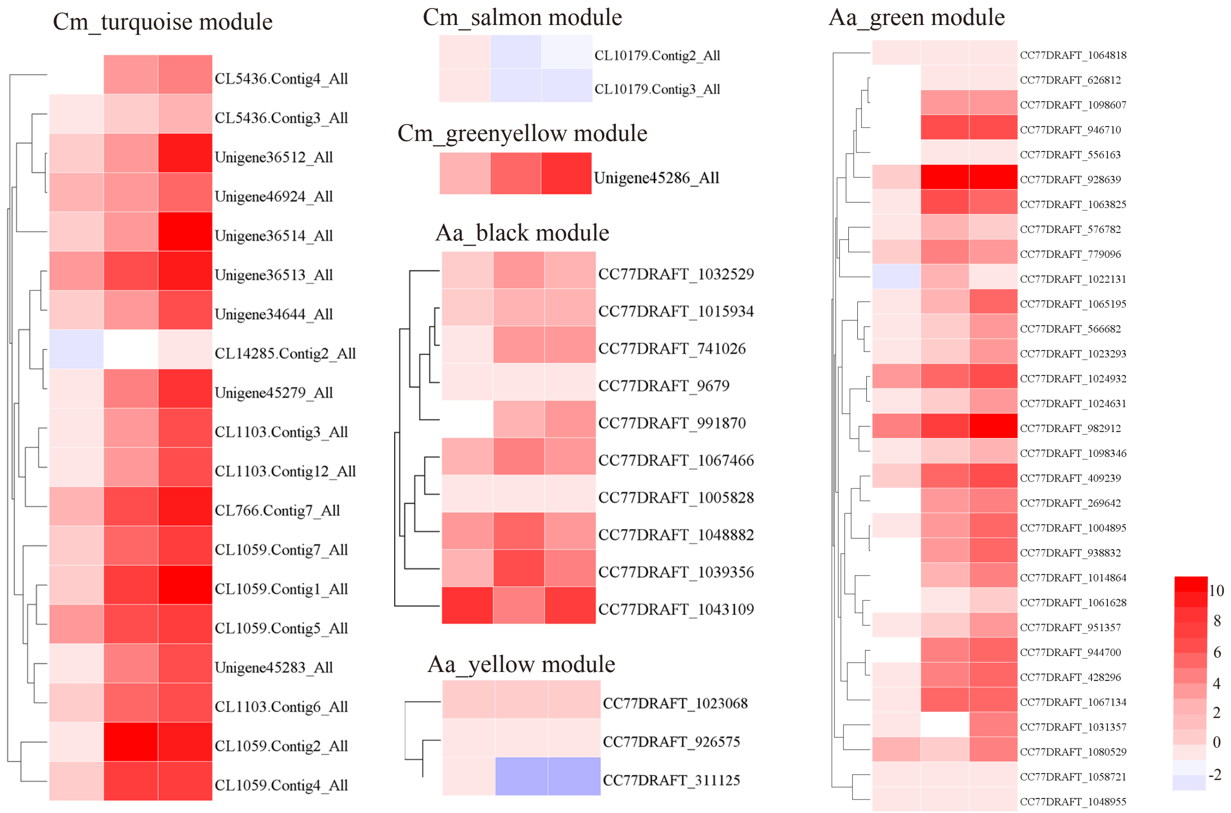

Cm_turquoise module
CL5436.Contig4_All
CL5436.Contig3_All
Unigene36512_All
Unigene46924_All
Unigene36514_All
Unigene36513_All
Unigene34644_All
CL14285.Contig2_All
Unigene45279_All
CL1103.Contig3_All
CL1103.Contig12_All
CL766.Contig7_All
CL1059.Contig7_All
CL1059.Contig1_All
CL1059.Contig5_All
Unigene45283_All
CL1103.Contig6_All
CL1059.Contig2_All
CL1059.Contig4_All
Cm_salmon module
CL10179.Contig2_All
CL10179.Contig3_All
Cm_greenyellow module
Unigene45286_All
Aa_black module
CC77DRAFT_1032529
CC77DRAFT_1015934
CC77DRAFT_741026
CC77DRAFT_9679
CC77DRAFT_991870
CC77DRAFT_1067466
CC77DRAFT_1005828
CC77DRAFT_1048882
CC77DRAFT_1039356
CC77DRAFT_1043109
Aa_yellow module
CC77DRAFT_1023068
CC77DRAFT_926575
CC77DRAFT_311125
Aa_green module
CC77DRAFT_1064818
CC77DRAFT_626812
CC77DRAFT_1098607
CC77DRAFT_946710
CC77DRAFT_556163
CC77DRAFT_928639
CC77DRAFT_1063825
CC77DRAFT_576782
CC77DRAFT_779096
CC77DRAFT_1022131
CC77DRAFT_1065195
CC77DRAFT_566682
CC77DRAFT_1023293
CC77DRAFT_1024932
CC77DRAFT_1024631
CC77DRAFT_982912
CC77DRAFT_1098346
CC77DRAFT_409239
CC77DRAFT_269642
CC77DRAFT_1004895
CC77DRAFT_938832
CC77DRAFT_1014864
CC77DRAFT_1061628
CC77DRAFT_951357
CC77DRAFT_944700
CC77DRAFT_428296
CC77DRAFT_1067134
CC77DRAFT_1031357
CC77DRAFT_1080529
CC77DRAFT_1058721
CC77DRAFT_1048955
10
8
6
4
2
0
-2
