# Supplementary figures and images for: Dual species dynamic transcripts reveal the interaction mechanisms between Chrysanthemum morifolium and Alternaria alternata
Source: BMC Genomics. 2021 Jul 9;22:523. doi: 10.1186/s12864-021-07709-9 (PMC8268330; doi:10.1186/s12864-021-07709-9)

## Ascorbic acid

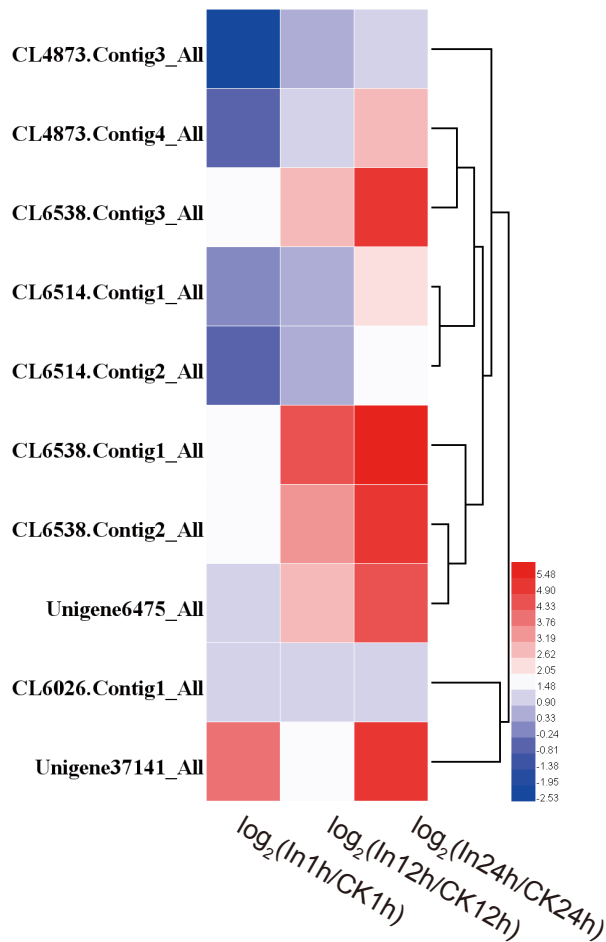

## Glutathione

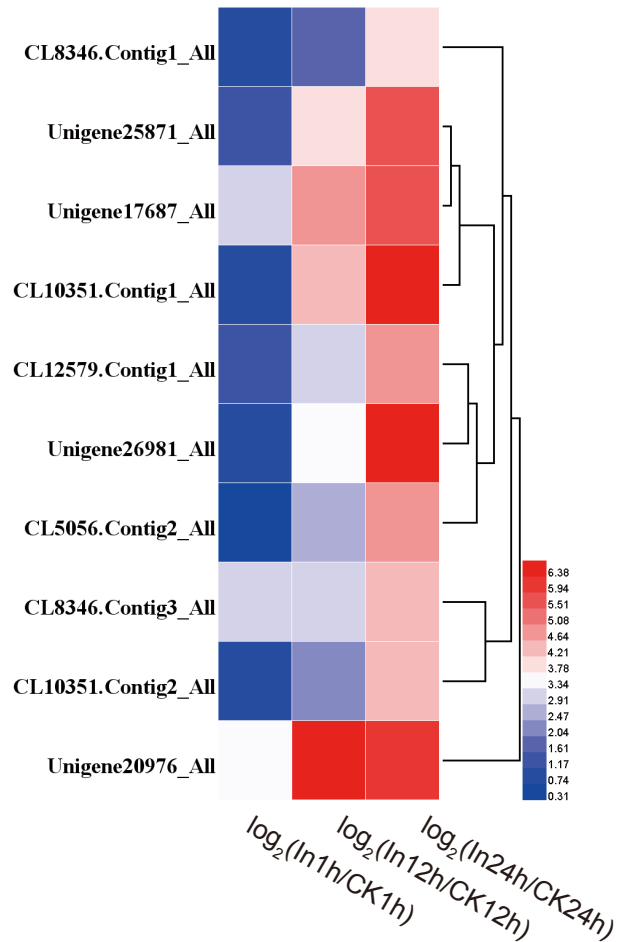

Supplement: Supplementary file 2 — Additional file 2: Figure S2 Heatmap of genes involved in ascorbic acid and glutathione synthesis. Expression values are presented as log2 fold-change value (red represents up-regulation; blue represents down-regulation). [file 12864_2021_7709_MOESM2_ESM.pdf]
