# Supplementary material for: Dual species dynamic transcripts reveal the interaction mechanisms between Chrysanthemum morifolium and Alternaria alternata
Source: BMC Genomics. 2021 Jul 9;22:523. doi: 10.1186/s12864-021-07709-9 (PMC8268330; doi:10.1186/s12864-021-07709-9)
Supplement: Supplementary file 3 — Additional file 3: Table S1 Primer sequences used in qRT-PCR for the validation of dual RNA-seq data. [file 12864_2021_7709_MOESM3_ESM.docx]

**Table S1** Primer sequences used in qRT-PCR for the validation of dual RNA-seq data.

| **Gene ID** | **Sequence (5'–3')** |
| --- | --- |
| CmEF1α-F  CmEF1α-R | TCAGGTCATCATCATGAACCA  AAGAGGTGGGTACTCAGCAAA |
| CL11098.Contig2_All-F | GAGCATAGGAAGAAAGTGCTG  CTAAACATAGCTTGACCAGGC |
| CL1653.Contig1_All-R | GAAGCAGTAGCAGTAGCAATCG  ATGTTGAGATCACCCACGGTTG |
| CL5572.Contig1_All | GGATAAACACTCCAGAAGAGCG  CTGAGCAGGGTTGTATGGATTC |
| Unigene47090_All | CCTCATTGCCAAAGCAGCTTGATC  GAACCTCATTTAGAGGCGTCAGAC |
| CL3907.Contig2_All | CTGGTCCATCATACGCCAATTC  GTGCTCTCCACCAACAACTTTG |
| CL11265.Contig3_All | GAAGCTCAGTTAAGAGTCGC  GTCCAAATGCAGAGAACGAC |
| CC77DRAFT_1036704 | CTGCAATATCACAGGTGACTG  CCTGTCCAGTTGTAGTTTGTC |
| CC77DRAFT_779096 | GAACACCACACTACGGATATG  AGTAACTTCCTGTTCCTTGGC |
| CC77DRAFT_598231 | CACAACTCTTTCCCGTACTTC  CAGTAACGCTTGGAGTTCTC |
| CC77DRAFT_950634 | GTGGCGATTACTACAATGTCG  GAAGATCTCGTTCGCTTCAAC |
| CC77DRAFT_945175 | CTATGGCTCTCAAGGAACTAG  GAACTGGCTCATCATTGAAGC |
| CC77DRAFT_1044312 | GTTCAGCGTTGGATCAGTAC  GGCTAGCTTGATCAGTGATG |
