# Supplementary material for: Dual species dynamic transcripts reveal the interaction mechanisms between Chrysanthemum morifolium and Alternaria alternata
Source: BMC Genomics. 2021 Jul 9;22:523. doi: 10.1186/s12864-021-07709-9 (PMC8268330; doi:10.1186/s12864-021-07709-9)
Supplement: Supplementary file 4 — Additional file 4: Table S2 Summary statistics of raw reads and clean reads used for mapping to reference genome of A. alternata. [file 12864_2021_7709_MOESM4_ESM.docx]

**Table S2** Summary statistics of raw reads and clean reads used for mapping to reference genome of *A. alternata*.

| **Sample** | **Total Raw**  **Reads (Mb)** | **Total Clean**  **Reads (Mb)** | **Total Clean**  **Bases (Gb)** | **Clean Reads Q20 (%)** | **Clean Reads Q30 (%)** | **Clean Reads**  **Ratio (%)** |
| --- | --- | --- | --- | --- | --- | --- |
| **Aa1h** | 114.94 | 106.92 | 10.69 | 97.31 | 89.88 | 93.02 |
|  | 114.94 | 107 | 10.7 | 97.44 | 90.27 | 93.09 |
|  | 112.44 | 104.06 | 10.41 | 97.27 | 89.72 | 92.54 |
| **Aa12h** | 114.94 | 110.31 | 11.03 | 98.09 | 91.57 | 95.97 |
|  | 114.94 | 110.38 | 11.04 | 98.15 | 91.8 | 96.03 |
|  | 114.88 | 110.71 | 11.07 | 98.25 | 92.12 | 96.37 |
| **Aa24h** | 114.94 | 110 | 11 | 98.09 | 91.62 | 95.7 |
|  | 112.44 | 108.19 | 10.82 | 98.31 | 92.44 | 96.22 |
|  | 114.94 | 110.22 | 11.02 | 98.21 | 92.09 | 95.89 |
| **Average** | 114.38 | 108.64 | 10.86 | 97.90 | 91.28 | 94.98 |
| **In1h** | 112.44 | 108.87 | 10.89 | 98.31 | 92.32 | 96.83 |
|  | 114.94 | 111.19 | 11.12 | 98.45 | 92.88 | 96.74 |
|  | 114.94 | 111.05 | 11.11 | 98.38 | 92.62 | 96.62 |
| **In12h** | 114.94 | 110.43 | 11.04 | 98.28 | 92.29 | 96.07 |
|  | 114.94 | 110.54 | 11.05 | 98.41 | 92.79 | 96.17 |
|  | 112.44 | 108.07 | 10.81 | 98.28 | 92.26 | 96.11 |
| **In24h** | 110.37 | 106.32 | 10.63 | 98.06 | 91.59 | 96.33 |
|  | 107.86 | 103.38 | 10.34 | 97.91 | 91.17 | 95.85 |
|  | 114.94 | 110.53 | 11.05 | 98.33 | 92.48 | 96.16 |
| **Average** | 113.09 | 108.93 | 10.89 | 98.27 | 92.27 | 96.32 |
