# Supplementary material for: Dual species dynamic transcripts reveal the interaction mechanisms between Chrysanthemum morifolium and Alternaria alternata
Source: BMC Genomics. 2021 Jul 9;22:523. doi: 10.1186/s12864-021-07709-9 (PMC8268330; doi:10.1186/s12864-021-07709-9)
Supplement: Supplementary file 5 — Additional file 5: Table S3 Summary statistics of chrysanthemum clean reads. [file 12864_2021_7709_MOESM5_ESM.docx]

**Table S3** Summary statistics of chrysanthemum clean reads.

| **Sample** | **Total Clean**  **Reads (Mb)** | **Total Clean**  **Bases (Gb)** | **Clean Reads Q20 (%)** | **Clean Reads Q30 (%)** | **Clean Reads**  **Ratio (%)** |
| --- | --- | --- | --- | --- | --- |
| **CK1h** | 107.38 | 10.74 | 97.62 | 90.69 | 100 |
|  | 107.14 | 10.71 | 97.67 | 90.94 | 100 |
|  | 107.53 | 10.75 | 97.61 | 90.69 | 100 |
| **CK12h** | 110.9 | 11.09 | 98.54 | 93.17 | 100 |
|  | 108.61 | 10.86 | 98.62 | 93.53 | 100 |
|  | 110 | 11 | 98.42 | 92.69 | 100 |
| **CK24h** | 110.74 | 11.07 | 98.53 | 93.15 | 100 |
|  | 108.48 | 10.85 | 98.47 | 92.86 | 100 |
|  | 108.1 | 10.81 | 98.46 | 92.86 | 100 |
| **Average** | 108.76 | 10.88 | 98.22 | 92.29 | 100 |
| **In1h** | 45.3 | 4.53 | 98.39 | 92.75 | 100 |
|  | 47.92 | 4.79 | 98.47 | 93.11 | 100 |
|  | 37.97 | 3.8 | 98.37 | 92.84 | 100 |
| **In12h** | 37.14 | 3.71 | 98.2 | 92.24 | 100 |
|  | 35.74 | 3.57 | 98.32 | 92.73 | 100 |
|  | 38.55 | 3.85 | 98.26 | 92.43 | 100 |
| **In24h** | 43.62 | 4.36 | 98.16 | 92.05 | 100 |
|  | 40.88 | 4.09 | 97.95 | 91.47 | 100 |
|  | 43.66 | 4.37 | 98.34 | 92.72 | 100 |
| **Average** | 41.20 | 4.12 | 98.27 | 92.48 | 100 |
