# Supplementary material for: Dual species dynamic transcripts reveal the interaction mechanisms between Chrysanthemum morifolium and Alternaria alternata
Source: BMC Genomics. 2021 Jul 9;22:523. doi: 10.1186/s12864-021-07709-9 (PMC8268330; doi:10.1186/s12864-021-07709-9)
Supplement: Supplementary file 6 — Additional file 6: Table S4 Summary of the assembly results of chrysanthemum. [file 12864_2021_7709_MOESM6_ESM.docx]

**Table S4** Summary of the assembly results of chrysanthemum.

| **Sample** | **Total Number** | **Total Length** | **Mean Length** | **N50** | **N70** | **N90** | **GC(%)** |
| --- | --- | --- | --- | --- | --- | --- | --- |
| **CK1h** | 51399 | 39924269 | 776 | 1081 | 715 | 366 | 40.54 |
|  | 60991 | 49686251 | 814 | 1157 | 757 | 376 | 40.47 |
|  | 53787 | 43655213 | 811 | 1160 | 746 | 370 | 40.65 |
| **CK12h** | 58005 | 45650884 | 787 | 1106 | 723 | 366 | 40.49 |
|  | 54057 | 41965352 | 776 | 1095 | 706 | 356 | 40.58 |
|  | 57455 | 46808244 | 814 | 1156 | 747 | 371 | 40.61 |
| **CK24h** | 52786 | 39322987 | 744 | 1016 | 685 | 356 | 40.45 |
|  | 47458 | 36032467 | 759 | 1064 | 692 | 347 | 40.7 |
|  | 55099 | 43194881 | 783 | 1098 | 721 | 362 | 40.48 |
| **Average** | 54560 | 42915616 | 785 | 1104 | 721 | 363 | 40.55 |
| **In1h** | 46920 | 34616729 | 737 | 1045 | 664 | 332 | 42.53 |
|  | 44278 | 29364412 | 663 | 869 | 598 | 322 | 42.07 |
|  | 37543 | 24138023 | 642 | 865 | 556 | 296 | 43.11 |
| **In12h** | 22195 | 14571366 | 656 | 901 | 556 | 298 | 45.37 |
|  | 24461 | 16613962 | 679 | 944 | 585 | 304 | 45.01 |
|  | 27980 | 20133835 | 719 | 1042 | 636 | 315 | 44.02 |
| **In24h** | 37071 | 27465624 | 740 | 1081 | 661 | 323 | 42.97 |
|  | 36335 | 25713984 | 707 | 1008 | 620 | 312 | 43.82 |
|  | 45808 | 33355888 | 728 | 1047 | 644 | 321 | 43.12 |
| **Average** | 35843 | 25108203 | 697 | 978 | 613 | 314 | 43.56 |
