# Supplementary material for: Dual species dynamic transcripts reveal the interaction mechanisms between Chrysanthemum morifolium and Alternaria alternata
Source: BMC Genomics. 2021 Jul 9;22:523. doi: 10.1186/s12864-021-07709-9 (PMC8268330; doi:10.1186/s12864-021-07709-9)
Supplement: Supplementary file 7 — Additional file 7: Table S5 Unigene annotation overview of chrysanthemum. [file 12864_2021_7709_MOESM7_ESM.docx]

**Table S5** Unigene annotation overview of chrysanthemum.

| **Values** | **NR** | **NT** | **Swissprot** | **KEGG** | **KOG** | **Pfam** | **GO** | **Overall** |
| --- | --- | --- | --- | --- | --- | --- | --- | --- |
| **Number** | 89,889 | 55,679 | 61,156 | 64,705 | 64,694 | 60,671 | 68,727 | 94,961 |
| **Percentage** | 72.62% | 44.98% | 49.41% | 52.27% | 52.26% | 49.01% | 55.52% | 76.72% |
