# Supplementary material for: Dual species dynamic transcripts reveal the interaction mechanisms between Chrysanthemum morifolium and Alternaria alternata
Source: BMC Genomics. 2021 Jul 9;22:523. doi: 10.1186/s12864-021-07709-9 (PMC8268330; doi:10.1186/s12864-021-07709-9)
Supplement: Supplementary file 8 — Additional file 8: Table S6 Summary of clean read mapping to A. alternata genomic database. [file 12864_2021_7709_MOESM8_ESM.docx]

**Table S6** Summary of clean read mapping to *A. alternata* genomic database.

| **Sample** | **Total Clean Reads (Mb)** | **Total Mapping(%)** | **Uniquely Mapping(%)** |
| --- | --- | --- | --- |
| **Aa1h** | 106.92 | 86.92 | 67.93 |
|  | 107 | 86.53 | 68.21 |
|  | 104.06 | 86.66 | 67.69 |
| **Aa12h** | 110.31 | 87.73 | 70.89 |
|  | 110.38 | 88.11 | 71.58 |
|  | 110.71 | 87.34 | 69.98 |
| **Aa24h** | 110 | 88.87 | 71.67 |
|  | 108.19 | 89.21 | 72.76 |
|  | 110.22 | 88.23 | 71.43 |
| **Average** | 108.64 | 87.73 | 70.24 |
| **In1h** | 108.87 | 58.4 | 47.5 |
|  | 111.19 | 56.9 | 46.7 |
|  | 111.05 | 65.81 | 53.62 |
| **In12h** | 110.43 | 66.37 | 54.11 |
|  | 110.54 | 67.67 | 55.09 |
|  | 108.07 | 64.33 | 52.29 |
| **In24h** | 106.32 | 58.97 | 47.23 |
|  | 103.38 | 60.46 | 48.69 |
|  | 110.53 | 60.5 | 49.33 |
| **Average** | 108.93 | 62.16 | 50.51 |
