# Supplementary material for: Dual species dynamic transcripts reveal the interaction mechanisms between Chrysanthemum morifolium and Alternaria alternata
Source: BMC Genomics. 2021 Jul 9;22:523. doi: 10.1186/s12864-021-07709-9 (PMC8268330; doi:10.1186/s12864-021-07709-9)
Supplement: Supplementary file 9 — Additional file 9: Table S7 Mapping rate of clean read mapped to A. alternata to chrysanthemum. [file 12864_2021_7709_MOESM9_ESM.docx]

**Table S7** Mapping rate of clean read mapped to *A. alternata* to chrysanthemum.

| **Sample** | | | **Total Mapping(%)** | | **Uniquely Mapping(%)** | |
| --- | --- | --- | --- | --- | --- | --- |
| **Aa1h_1** | | 0.10 | | | 0.07 | |
| **Aa1h_2** | | 0.14 | | | 0.09 | |
| **Aa1h_3** | | 0.13 | | | 0.09 | |
| **Aa12h_1** | | 0.13 | | | 0.08 | |
| **Aa12h_2** | | 0.12 | | | 0.07 | |
| **Aa12h_3** | | 0.15 | | | 0.10 | |
| **Aa24h_1** | | 0.10 | | | 0.06 | |
| **Aa24h_2** | | 0.17 | | | 0.10 | |
| **Aa24h_3** | | 0.15 | | | 0.10 | |
| **In1h_1** | | 0.10 | | | 0.06 | |
| **In1h_2** | | 0.19 | | | 0.14 | |
| **In1h_3** | | 0.08 | | | 0.05 | |
| **In12h_1** | | 0.12 | | | 0.08 | |
| **In12h_2** | | 0.08 | | | 0.05 | |
| **In12h_3** | | 0.14 | | | 0.08 | |
| **In24h_1** | | 0.13 | | | 0.07 | |
| **In24h_2** | | 0.06 | | | 0.04 | |
| **In24h_3** | | 0.07 | | | 0.04 | |
|  |  | | |  | |  |
