# Supplementary material for: Dual species dynamic transcripts reveal the interaction mechanisms between Chrysanthemum morifolium and Alternaria alternata
Source: BMC Genomics. 2021 Jul 9;22:523. doi: 10.1186/s12864-021-07709-9 (PMC8268330; doi:10.1186/s12864-021-07709-9)
Supplement: Supplementary file 10 — Additional file 10:Table S8 Results of KEGG pathway enrichment analysis of chrysanthemum. [file 12864_2021_7709_MOESM10_ESM.docx]

**Table S8** Results of kyoto encyclopedia of genes and genomes (KEGG) pathway enrichment analysis of chrysanthemum.

| **Pathway ID** | **Pathway Name** | **Number of genes with pathway annotation** | **Number of DEGs at each individual time point** | | |
| --- | --- | --- | --- | --- | --- |
|  |  |  | **1 HPI** | **12 HPI** | **24 HPI** |
| ko04626 | Plant-pathogen interaction | 2100 | 509 | 1337 | 986 |
| ko04075 | Plant hormone signal transduction | 1725 | 375 | 1215 | 774 |
| ko04016 | MAPK signaling pathway - plant | 1647 | 409 | 1077 | 776 |
| ko01200 | Carbon metabolism | 1556 | 372 | 1033 | 812 |
| ko04141 | Protein processing in endoplasmic reticulum | 1788 | 318 | 1022 | 753 |
| ko01230 | Biosynthesis of amino acids | 1505 | 297 | 1004 | 716 |
| ko00940 | Phenylpropanoid biosynthesis | 1196 | 427 | 849 | 714 |
| ko04144 | Endocytosis | 1588 | 285 | 935 | 644 |
| ko00500 | Starch and sucrose metabolism | 1134 | 250 | 737 | 489 |
| ko04120 | Ubiquitin-mediated proteolysis | 992 | 156 | 606 | 351 |
| ko00270 | Cysteine and methionine metabolism | 570 | 130 | 409 | 268 |
| ko00460 | Cyanoamino acid metabolism | 522 | 141 | 345 | 257 |
| ko04145 | Phagosome | 565 | 123 | 324 | 233 |
| ko03030 | DNA replication | 541 | 69 | 386 | 181 |
| ko02010 | ABC transporters | 437 | 108 | 283 | 228 |
| ko00260 | Glycine, serine, and threonine metabolism | 405 | 110 | 297 | 210 |
| ko00592 | alpha-Linolenic acid metabolism | 384 | 148 | 238 | 204 |
| ko00480 | Glutathione metabolism | 421 | 90 | 270 | 199 |
| ko00380 | Tryptophan metabolism | 342 | 114 | 238 | 175 |
| ko00360 | Phenylalanine metabolism | 313 | 129 | 226 | 192 |
| ko00906 | Carotenoid biosynthesis | 269 | 110 | 211 | 145 |
| ko00900 | Terpenoid backbone biosynthesis | 315 | 77 | 199 | 146 |
| ko00310 | Lysine degradation | 247 | 54 | 155 | 127 |
| ko00945 | Stilbenoid, diarylheptanoid, and gingerol biosynthesis | 171 | 63 | 125 | 109 |
| ko00330 | Arginine and proline metabolism | 322 | 96 | 214 | 163 |
| ko00909 | Sesquiterpenoid and triterpenoid biosynthesis | 183 | 55 | 118 | 87 |
| ko00591 | Linoleic acid metabolism | 127 | 59 | 88 | 77 |
| ko00100 | Steroid biosynthesis | 172 | 33 | 121 | 69 |
| ko04130 | SNARE interactions in vesicular transport | 167 | 30 | 103 | 53 |
| ko00072 | Synthesis and degradation of ketone bodies | 63 | 15 | 34 | 36 |
