# Supplementary material for: Dual species dynamic transcripts reveal the interaction mechanisms between Chrysanthemum morifolium and Alternaria alternata
Source: BMC Genomics. 2021 Jul 9;22:523. doi: 10.1186/s12864-021-07709-9 (PMC8268330; doi:10.1186/s12864-021-07709-9)
Supplement: Supplementary file 11 — Additional file 11: Table S9 Results of KEGG pathway enrichment analysis of A. alternata. [file 12864_2021_7709_MOESM11_ESM.docx]

**Table S9** Results of Kyoto Encyclopedia of Genes and Genomes (KEGG) pathway enrichment analysis of *A. alternata*.

| **Pathway ID** | **Pathway Name** | **Number of genes with pathway annotation** | **Number of DEGs at each individual time point** | | |
| --- | --- | --- | --- | --- | --- |
|  |  |  | **1HPI** | **12HPI** | **24HPI** |
| ko01130 | Biosynthesis of antibiotics | 833 | 312 | 430 | 390 |
| ko04011 | MAPK signaling pathway - yeast | 585 | 154 | 218 | 196 |
| ko00520 | Amino sugar and nucleotide sugar metabolism | 395 | 103 | 161 | 159 |
| ko00260 | Glycine, serine and threonine metabolism | 262 | 107 | 124 | 127 |
| ko01200 | Carbon metabolism | 239 | 95 | 129 | 121 |
| ko04146 | Peroxisome | 194 | 83 | 118 | 116 |
| ko00500 | Starch and sucrose metabolism | 226 | 87 | 120 | 113 |
| ko01230 | Biosynthesis of amino acids | 252 | 87 | 112 | 107 |
| ko00230 | Purine metabolism | 260 | 78 | 113 | 106 |
| ko00350 | Tyrosine metabolism | 180 | 84 | 97 | 103 |
| ko03013 | RNA transport | 277 | 69 | 90 | 96 |
| ko00010 | Glycolysis / Gluconeogenesis | 169 | 79 | 101 | 94 |
| ko00380 | Tryptophan metabolism | 188 | 77 | 100 | 94 |
| ko04141 | Protein processing in endoplasmic reticulum | 230 | 63 | 79 | 94 |
| ko00040 | Pentose and glucuronate interconversions | 146 | 73 | 96 | 93 |
| ko00564 | Glycerophospholipid metabolism | 200 | 61 | 101 | 93 |
| ko00071 | Fatty acid degradation | 144 | 66 | 86 | 85 |
| ko00620 | Pyruvate metabolism | 154 | 66 | 88 | 82 |
| ko04144 | Endocytosis | 279 | 71 | 96 | 82 |
| ko00051 | Fructose and mannose metabolism | 123 | 69 | 77 | 76 |
